# Supplementary figures and images for: The Nutritional Content of Rescued Food Conveyed by a Food Aid Organization
Source: Int J Environ Res Public Health. 2021 Nov 20;18(22):12212. doi: 10.3390/ijerph182212212 (PMC8624580; doi:10.3390/ijerph182212212)

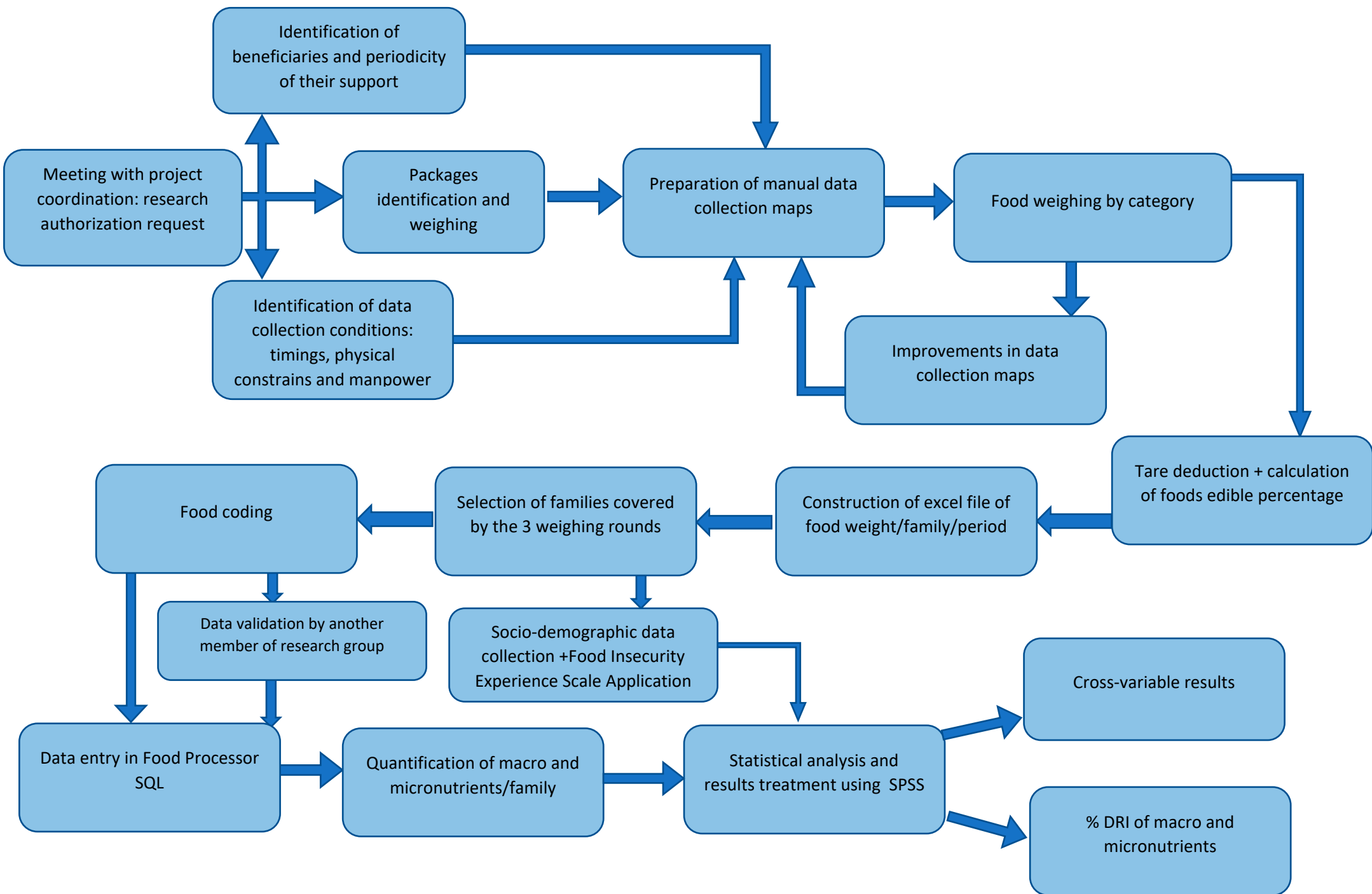

Supplement: Supplementary file 1 [file ijerph-18-12212-s001.zip › ijerph-1270364-supplementary.pdf]
